# 2,4,6,7-Tetra­methyl-3-phenyl­sulfinyl-1-benzofuran

**DOI:** 10.1107/S1600536808015031

**Published:** 2008-05-24

**Authors:** Hong Dae Choi, Pil Ja Seo, Byeng Wha Son, Uk Lee

**Affiliations:** a Department of Chemistry, Dongeui University, San 24 Kaya-dong, Busanjin-gu, Busan 614-714, Republic of Korea; b Department of Chemistry, Pukyong National University, 599-1 Daeyeon 3-dong, Nam-gu, Busan 608-737, Republic of Korea

## Abstract

In the title compound, C_18_H_18_O_2_S, the O atom and the phenyl group of the phenyl­sulfinyl substituent lie on opposite sides of the planar benzofuran fragment. The phenyl ring is nearly perpendicular to the benzofuran system [88.56 (7)°] and is tilted slightly towards it. Molecules form pseudo-helices along the *a* axis. The crystal structure is stabilized by a C—H⋯π inter­action between a methyl H atom and the phenyl ring of the phenyl­sulfinyl substituent, and by intra- and inter­molecular C—H⋯O inter­actions.

## Related literature

For details of the pharmacological properties of benzofuran compounds, see: Howlett *et al.* (1999 ▶); Ward (1997 ▶). For the structures of other benzofuran derivatives, see: Choi *et al.* (2007 ▶, 2008 ▶).
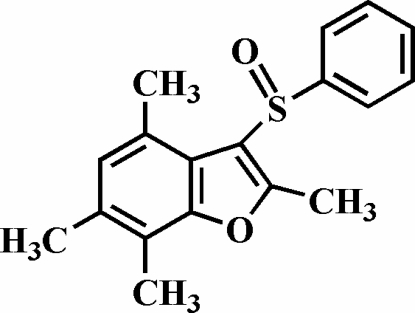


## Experimental

### 

#### Crystal data

C_18_H_18_O_2_S
*M* *_r_* = 298.38
Orthorhombic, 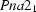

*a* = 12.0402 (6) Å
*b* = 19.673 (1) Å
*c* = 6.4399 (3) Å
*V* = 1525.40 (13) Å^3^
*Z* = 4
Mo *K*α radiation
μ = 0.21 mm^−1^
*T* = 173 (2) K
0.40 × 0.40 × 0.30 mm

#### Data collection

Bruker SMART CCD diffractometer
Absorption correction: none
9082 measured reflections
2486 independent reflections
2339 reflections with *I* > 2σ(*I*)
*R*_int_ = 0.102

#### Refinement

*R*[*F* ^2^ > 2σ(*F* ^2^)] = 0.051
*wR*(*F* ^2^) = 0.138
*S* = 1.08
2486 reflections
194 parameters
1 restraint
H-atom parameters constrained
Δρ_max_ = 0.51 e Å^−3^
Δρ_min_ = −0.43 e Å^−3^

### 

Data collection: *SMART* (Bruker, 2001 ▶); cell refinement: *SAINT* (Bruker, 2001 ▶); data reduction: *SAINT*; program(s) used to solve structure: *SHELXS97* (Sheldrick, 2008 ▶); program(s) used to refine structure: *SHELXL97* (Sheldrick, 2008 ▶); molecular graphics: *ORTEP-3* (Farrugia, 1997 ▶) and *DIAMOND* (Brandenburg, 1998 ▶); software used to prepare material for publication: *SHELXL97*.

## Supplementary Material

Crystal structure: contains datablocks global, I. DOI: 10.1107/S1600536808015031/fl2196sup1.cif

Structure factors: contains datablocks I. DOI: 10.1107/S1600536808015031/fl2196Isup2.hkl

Additional supplementary materials:  crystallographic information; 3D view; checkCIF report

## Figures and Tables

**Table 1:** Hydrogen-bond geometry (Å, °)

*D*—H⋯*A*	*D*—H	H⋯*A*	*D*⋯*A*	*D*—H⋯*A*
C17—H17*A*⋯*Cg*^i^	0.98	2.68	3.565 (5)	151
C10—H10⋯O2^i^	0.95	2.48	3.306 (4)	146
C15—H15*B*⋯O2	0.98	2.38	3.248 (4)	147

Symmetry code: (i) 
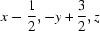
. *Cg* is the centroid of the C9–C14 phenyl ring.

**Fig. 1.**
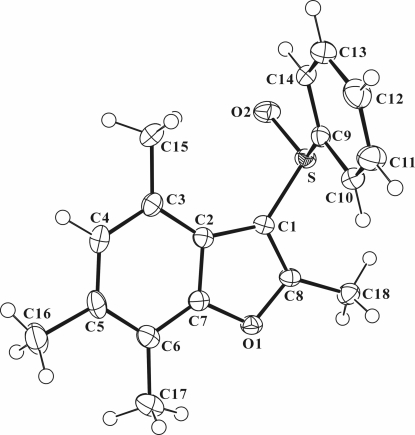
The molecular structure of the title compound, showing displacement ellipsoids drawn at the 50% probability level.

**Fig. 2.**
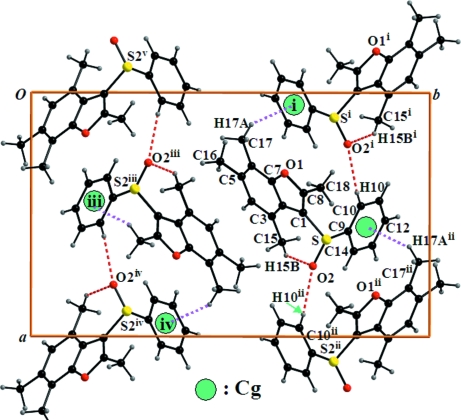
View of the structure projected on the ab plane. C—H···π and C—H···O interactions are shown as dotted lines. Cg denotes the ring centroid. [Symmetry code: (i)x-1/2, -y+3/2, z; (ii) x+1/2, -y+3/2, z; (iii) -x+1, -y+1, z+1/2; (iv) -x+3/2, y-1/2, z-1/2; (iv) -x+1/2, y-1/2, z+1/2.]

## supplementary crystallographic information

### Comment

Benzofuran ring systems have attracted considerable interest because of their various pharmacological properties (Howlett *et al.*, 1999; Ward, 1997). This work is related to earlier communications on the synthesis and structure of similar benzofuran analogues (Choi *et al.*, 2007, 2008)

In the title commmpound, the benzofuran unit is essentially planar, with a mean deviation of 0.008 Å from the least-squares plane defined by the nine constituent atoms (Fig. 1). The phenyl ring (C9-C14) is almost perpendicular to the plane of the benzofuran system [88.56 (7)°] and is tilted slightly towards it.

The title commpound crystallized in the non-centrosymmetric space group Pna21 in spite of having no asymmetric carbon atoms. The space group was caused by a right handed pseudo-helix along the a axis.  In addition, the molecular packing (Fig. 2) is stabilized by a C—H···π interaction between a methyl H atom and the phenyl ring of the phenylsulfinyl substituent, with a C17—17A···Cg^i^ separation of 3.565 (5) Å (Fig. 2 and Table1; Cg is the centroid of C9-C14 phenyl ring). The molecular packing  is further stabilized by intra-  and intermolecular C—H···O interactions (Fig. 2 and Table 1: symmetry code as in Fig. 2).

### Experimental

3-Chloroperoxybenzoic acid (77%, 190 mg, 0.85 mmol) was added in small portions to a stirred solution of 2,4,6,7-tetramethyl-3-phenylsulfanyl-1-benzofuran (226 mg, 0.8 mmol) in dichloromethane (20 ml) at 273 K. After being stirred at room temperature for 2h, the mixture was washed with a saturated soution of sodium bicarbonate and the organic layer was separated, dried over magnesium sulfate, filtered and concentrated in vacuum. The residue was purified by column chromatography (hexane-ethyl acetate, 2:1 v/v) to afford the title compound as a colorless solid [yield 80%, m.p. 407-408 K; R_f_ = 0.54 (hexane-ethyl acetate, 2:1 v/v)]. Single crystals suitable for X-ray diffraction were prepared by slow evaporation from  acetone at room temperature. Spectroscopic analysis: ^1^H NMR (CDCl_3_, 400 MHz) δ 2.13 (s, 3H), 2.29 (s, 3H), 2.37 (s, 3H), 2.71 (s, 3H), 6.77 (s, 1H), 7.39-7.45 (m, 3H), 7.48-7.51 (m, 2H); EI-MS 298 [M^+^].

### Refinement

All H atoms were positioned geometrically and refined using a riding model, with C—H = 0.95 Å for aromatic H atoms and 0.98 Å for methyl H atoms, respectively, and with Uiso(H) = 1.2Ueq(C) for aromatic H atoms and 1.5Ueq(C) for methyl H atoms. Friedel pairs were merged at final refinement.

### Crystal data

**Table:**

C_18_H_18_O_2_S	*F*_000_ = 632
*M**_r_* = 298.38	*D*_x_ = 1.299 Mg m^−^^3^
Orthorhombic, *P**n**a*2_1_	Mo *K*α radiation λ = 0.71073 Å
Hall symbol: p_2c_-2n	Cell parameters from 5414 reflections
*a* = 12.0402 (6) Å	θ = 2.7–28.2º
*b* = 19.673 (1) Å	µ = 0.21 mm^−^^1^
*c* = 6.4399 (3) Å	*T* = 173 (2) K
*V* = 1525.40 (13) Å^3^	Block, colorless
*Z* = 4	0.40 × 0.40 × 0.30 mm

### Data collection

**Table:**

Bruker SMART CCD diffractometer	2486 independent reflections
Radiation source: fine-focus sealed tube	2339 reflections with *I* > 2σ(*I*)
Monochromator: graphite	*R*_int_ = 0.102
Detector resolution: 10.0 pixels mm^-1^	θ_max_ = 27.0º
*T* = 173(2) K	θ_min_ = 2.7º
φ and ω scans	*h* = −14→15
Absorption correction: none	*k* = −25→24
9082 measured reflections	*l* = −8→4

### Refinement

**Table:**

Refinement on *F*^2^	Secondary atom site location: difference Fourier map
Least-squares matrix: full	Hydrogen site location: inferred from neighbouring sites
*R*[*F*^2^ > 2σ(*F*^2^)] = 0.051	H-atom parameters constrained
*wR*(*F*^2^) = 0.138	*w* = 1/[σ^2^(*F*_o_^2^) + (0.0864*P*)^2^ + 0.5202*P*] where *P* = (*F*_o_^2^ + 2*F*_c_^2^)/3
*S* = 1.09	(Δ/σ)_max_ < 0.001
2486 reflections	Δρ_max_ = 0.51 e Å^−^^3^
194 parameters	Δρ_min_ = −0.43 e Å^−^^3^
1 restraint	Extinction correction: none
Primary atom site location: structure-invariant direct methods	

### Special details

**Table:**

Geometry. All esds (except the esd in the dihedral angle between two l.s. planes) are estimated using the full covariance matrix. The cell esds are taken into account individually in the estimation of esds in distances, angles and torsion angles; correlations between esds in cell parameters are only used when they are defined by crystal symmetry. An approximate (isotropic) treatment of cell esds is used for estimating esds involving l.s. planes.
Refinement. Refinement of F^2^ against ALL reflections. The weighted R-factor wR and goodness of fit S are based on F^2^, conventional R-factors R are based on F, with F set to zero for negative F^2^. The threshold expression of F^2^ > 2sigma(F^2^) is used only for calculating R-factors(gt) etc. and is not relevant to the choice of reflections for refinement. R-factors based on F^2^ are statistically about twice as large as those based on F, and R- factors based on ALL data will be even larger.

### Fractional atomic coordinates and isotropic or equivalent isotropic displacement parameters (Å^2^)

**Table:**

	*x*	*y*	*z*	*U*_iso_*/*U*_eq_	
S	0.60317 (5)	0.74039 (3)	0.74685 (15)	0.0220 (2)	
O1	0.33116 (17)	0.63540 (10)	0.8280 (4)	0.0229 (5)	
O2	0.71121 (16)	0.70681 (11)	0.6963 (4)	0.0308 (6)	
C1	0.4924 (2)	0.68198 (13)	0.7252 (5)	0.0200 (6)	
C2	0.4634 (2)	0.63018 (13)	0.5731 (5)	0.0199 (6)	
C3	0.5073 (3)	0.60387 (14)	0.3879 (6)	0.0237 (6)	
C4	0.4461 (3)	0.55262 (15)	0.2916 (5)	0.0274 (7)	
H4	0.4744	0.5336	0.1668	0.033*	
C5	0.3448 (3)	0.52741 (14)	0.3689 (6)	0.0277 (7)	
C6	0.3011 (2)	0.55259 (14)	0.5539 (6)	0.0245 (7)	
C7	0.3638 (2)	0.60360 (14)	0.6471 (5)	0.0207 (6)	
C8	0.4118 (2)	0.68331 (14)	0.8720 (5)	0.0205 (6)	
C9	0.5685 (2)	0.79514 (14)	0.5322 (5)	0.0204 (6)	
C10	0.4647 (3)	0.82691 (15)	0.5279 (6)	0.0274 (7)	
H10	0.4101	0.8171	0.6300	0.033*	
C11	0.4437 (3)	0.87313 (16)	0.3706 (7)	0.0339 (8)	
H11	0.3734	0.8950	0.3640	0.041*	
C12	0.5237 (3)	0.88799 (16)	0.2224 (7)	0.0344 (8)	
H12	0.5081	0.9202	0.1163	0.041*	
C13	0.6266 (3)	0.85595 (15)	0.2287 (7)	0.0290 (7)	
H13	0.6811	0.8658	0.1264	0.035*	
C14	0.6494 (2)	0.80925 (14)	0.3858 (6)	0.0235 (7)	
H14	0.7196	0.7874	0.3923	0.028*	
C15	0.6131 (3)	0.62939 (17)	0.2903 (6)	0.0281 (8)	
H15A	0.6436	0.5943	0.1986	0.042*	
H15B	0.6672	0.6400	0.3994	0.042*	
H15C	0.5975	0.6705	0.2095	0.042*	
C16	0.2833 (3)	0.47364 (15)	0.2454 (9)	0.0399 (9)	
H16A	0.2898	0.4297	0.3160	0.060*	
H16B	0.3156	0.4703	0.1061	0.060*	
H16C	0.2047	0.4862	0.2344	0.060*	
C17	0.1937 (3)	0.52894 (17)	0.6488 (7)	0.0337 (8)	
H17A	0.1314	0.5521	0.5812	0.051*	
H17B	0.1935	0.5396	0.7975	0.051*	
H17C	0.1862	0.4797	0.6296	0.051*	
C18	0.3902 (3)	0.72565 (17)	1.0569 (6)	0.0270 (7)	
H18A	0.4556	0.7536	1.0868	0.041*	
H18B	0.3743	0.6963	1.1761	0.041*	
H18C	0.3262	0.7552	1.0304	0.041*	

### Atomic displacement parameters (Å^2^)

**Table:**

	*U*^11^	*U*^22^	*U*^33^	*U*^12^	*U*^13^	*U*^23^
S	0.0194 (3)	0.0245 (3)	0.0222 (4)	−0.0027 (2)	−0.0002 (3)	−0.0011 (4)
O1	0.0215 (10)	0.0254 (10)	0.0219 (12)	−0.0032 (8)	0.0032 (9)	0.0003 (9)
O2	0.0200 (10)	0.0354 (11)	0.0369 (16)	0.0011 (8)	−0.0010 (10)	0.0046 (10)
C1	0.0186 (12)	0.0206 (12)	0.0208 (17)	−0.0016 (9)	−0.0004 (12)	0.0020 (13)
C2	0.0207 (14)	0.0170 (12)	0.0219 (16)	0.0013 (10)	−0.0010 (13)	0.0037 (12)
C3	0.0248 (14)	0.0217 (14)	0.0245 (17)	0.0062 (10)	0.0002 (13)	0.0007 (13)
C4	0.0332 (16)	0.0252 (14)	0.0239 (19)	0.0066 (11)	−0.0020 (14)	−0.0058 (12)
C5	0.0322 (17)	0.0164 (12)	0.034 (2)	0.0029 (11)	−0.0089 (15)	−0.0041 (14)
C6	0.0234 (14)	0.0177 (12)	0.0323 (19)	0.0013 (10)	−0.0036 (14)	0.0047 (13)
C7	0.0201 (12)	0.0185 (12)	0.0234 (17)	0.0022 (10)	0.0002 (13)	0.0037 (12)
C8	0.0194 (13)	0.0212 (13)	0.0210 (16)	−0.0017 (10)	0.0018 (13)	0.0036 (12)
C9	0.0205 (13)	0.0170 (13)	0.0237 (16)	0.0009 (10)	−0.0002 (12)	−0.0011 (12)
C10	0.0232 (15)	0.0250 (14)	0.0340 (19)	−0.0003 (11)	0.0061 (14)	−0.0015 (13)
C11	0.0251 (16)	0.0282 (16)	0.048 (2)	0.0039 (11)	0.0034 (16)	0.0082 (16)
C12	0.0428 (18)	0.0261 (14)	0.034 (2)	−0.0007 (12)	0.0011 (17)	0.0064 (16)
C13	0.0305 (15)	0.0271 (14)	0.029 (2)	−0.0045 (11)	0.0062 (17)	0.0021 (16)
C14	0.0204 (14)	0.0217 (12)	0.0282 (18)	0.0002 (10)	0.0054 (13)	−0.0048 (13)
C15	0.0266 (15)	0.0333 (15)	0.025 (2)	0.0041 (11)	0.0058 (14)	−0.0023 (13)
C16	0.0422 (18)	0.0277 (15)	0.050 (2)	−0.0004 (12)	−0.007 (2)	−0.012 (2)
C17	0.0294 (16)	0.0307 (16)	0.041 (2)	−0.0080 (12)	−0.0016 (16)	0.0059 (16)
C18	0.0251 (16)	0.0323 (15)	0.0236 (18)	−0.0001 (12)	0.0020 (14)	−0.0031 (14)

### Geometric parameters (Å, °)

**Table:**

S—O2	1.495 (2)	C10—H10	0.9500
S—C1	1.766 (3)	C11—C12	1.387 (5)
S—C9	1.801 (3)	C11—H11	0.9500
O1—C7	1.379 (4)	C12—C13	1.391 (5)
O1—C8	1.383 (3)	C12—H12	0.9500
C1—C8	1.355 (4)	C13—C14	1.394 (5)
C1—C2	1.456 (4)	C13—H13	0.9500
C2—C7	1.393 (4)	C14—H14	0.9500
C2—C3	1.403 (5)	C15—H15A	0.9800
C3—C4	1.394 (4)	C15—H15B	0.9800
C3—C15	1.507 (4)	C15—H15C	0.9800
C4—C5	1.408 (5)	C16—H16A	0.9800
C4—H4	0.9500	C16—H16B	0.9800
C5—C6	1.393 (5)	C16—H16C	0.9800
C5—C16	1.516 (5)	C17—H17A	0.9800
C6—C7	1.392 (4)	C17—H17B	0.9800
C6—C17	1.504 (5)	C17—H17C	0.9800
C8—C18	1.476 (5)	C18—H18A	0.9800
C9—C14	1.383 (5)	C18—H18B	0.9800
C9—C10	1.398 (4)	C18—H18C	0.9800
C10—C11	1.385 (5)		
			
O2—S—C1	110.63 (13)	C10—C11—H11	119.5
O2—S—C9	107.37 (15)	C12—C11—H11	119.5
C1—S—C9	98.84 (14)	C11—C12—C13	120.2 (3)
C7—O1—C8	106.4 (2)	C11—C12—H12	119.9
C8—C1—C2	108.1 (2)	C13—C12—H12	119.9
C8—C1—S	118.2 (2)	C12—C13—C14	119.7 (3)
C2—C1—S	133.6 (2)	C12—C13—H13	120.2
C7—C2—C3	118.5 (3)	C14—C13—H13	120.2
C7—C2—C1	103.8 (3)	C9—C14—C13	119.2 (3)
C3—C2—C1	137.7 (3)	C9—C14—H14	120.4
C4—C3—C2	116.5 (3)	C13—C14—H14	120.4
C4—C3—C15	120.1 (3)	C3—C15—H15A	109.5
C2—C3—C15	123.4 (3)	C3—C15—H15B	109.5
C3—C4—C5	123.7 (3)	H15A—C15—H15B	109.5
C3—C4—H4	118.1	C3—C15—H15C	109.5
C5—C4—H4	118.1	H15A—C15—H15C	109.5
C6—C5—C4	120.3 (3)	H15B—C15—H15C	109.5
C6—C5—C16	120.8 (3)	C5—C16—H16A	109.5
C4—C5—C16	118.9 (3)	C5—C16—H16B	109.5
C7—C6—C5	114.9 (3)	H16A—C16—H16B	109.5
C7—C6—C17	120.9 (3)	C5—C16—H16C	109.5
C5—C6—C17	124.2 (3)	H16A—C16—H16C	109.5
O1—C7—C6	122.5 (3)	H16B—C16—H16C	109.5
O1—C7—C2	111.3 (3)	C6—C17—H17A	109.5
C6—C7—C2	126.2 (3)	C6—C17—H17B	109.5
C1—C8—O1	110.3 (3)	H17A—C17—H17B	109.5
C1—C8—C18	134.4 (3)	C6—C17—H17C	109.5
O1—C8—C18	115.2 (3)	H17A—C17—H17C	109.5
C14—C9—C10	121.7 (3)	H17B—C17—H17C	109.5
C14—C9—S	118.7 (2)	C8—C18—H18A	109.5
C10—C9—S	119.3 (3)	C8—C18—H18B	109.5
C11—C10—C9	118.1 (3)	H18A—C18—H18B	109.5
C11—C10—H10	120.9	C8—C18—H18C	109.5
C9—C10—H10	120.9	H18A—C18—H18C	109.5
C10—C11—C12	121.0 (3)	H18B—C18—H18C	109.5
			
O2—S—C1—C8	138.4 (2)	C5—C6—C7—C2	−0.3 (4)
C9—S—C1—C8	−109.2 (3)	C17—C6—C7—C2	−179.2 (3)
O2—S—C1—C2	−43.7 (3)	C3—C2—C7—O1	−179.1 (2)
C9—S—C1—C2	68.7 (3)	C1—C2—C7—O1	0.4 (3)
C8—C1—C2—C7	−0.5 (3)	C3—C2—C7—C6	−0.4 (5)
S—C1—C2—C7	−178.5 (2)	C1—C2—C7—C6	179.2 (3)
C8—C1—C2—C3	179.0 (3)	C2—C1—C8—O1	0.3 (3)
S—C1—C2—C3	0.9 (6)	S—C1—C8—O1	178.72 (19)
C7—C2—C3—C4	0.2 (4)	C2—C1—C8—C18	−176.4 (3)
C1—C2—C3—C4	−179.2 (3)	S—C1—C8—C18	2.0 (5)
C7—C2—C3—C15	178.8 (3)	C7—O1—C8—C1	0.0 (3)
C1—C2—C3—C15	−0.6 (6)	C7—O1—C8—C18	177.4 (3)
C2—C3—C4—C5	0.6 (5)	O2—S—C9—C14	−13.1 (3)
C15—C3—C4—C5	−178.0 (3)	C1—S—C9—C14	−128.1 (3)
C3—C4—C5—C6	−1.4 (5)	O2—S—C9—C10	172.6 (2)
C3—C4—C5—C16	177.4 (3)	C1—S—C9—C10	57.6 (3)
C4—C5—C6—C7	1.2 (4)	C14—C9—C10—C11	0.5 (5)
C16—C5—C6—C7	−177.6 (3)	S—C9—C10—C11	174.7 (3)
C4—C5—C6—C17	−180.0 (3)	C9—C10—C11—C12	−0.6 (5)
C16—C5—C6—C17	1.3 (5)	C10—C11—C12—C13	0.6 (6)
C8—O1—C7—C6	−179.1 (3)	C11—C12—C13—C14	−0.6 (5)
C8—O1—C7—C2	−0.3 (3)	C10—C9—C14—C13	−0.5 (5)
C5—C6—C7—O1	178.3 (3)	S—C9—C14—C13	−174.7 (2)
C17—C6—C7—O1	−0.6 (5)	C12—C13—C14—C9	0.6 (5)

### Hydrogen-bond geometry (Å, °)

**Table:**

*D*—H···*A*	*D*—H	H···*A*	*D*···*A*	*D*—H···*A*
C17—H17A···Cg^i^	0.98	2.68	3.565 (5)	151
C10—H10···O2^i^	0.95	2.48	3.306 (4)	146
C15—H15B···O2	0.98	2.38	3.248 (4)	147

Symmetry codes: (i) *x*−1/2, −*y*+3/2, *z*.
